# ^1^H NMR Study of the Lipid Composition, Oxidative and Hydrolytic Status of the Covering Oils of Canned Sardines After Long-Term Storage

**DOI:** 10.3390/foods14091589

**Published:** 2025-04-30

**Authors:** Encarnacion Goicoechea-Oses

**Affiliations:** Food Technology, Faculty of Pharmacy, Lascaray Research Center, University of the Basque Country (UPV/EHU), 01006 Vitoria-Gasteiz, Spain; encarnacion.goicoechea@ehu.eus; Tel.: +34-945013083

**Keywords:** covering liquid, vegetable oil, canned sardines, tinned fish, proton nuclear magnetic resonance spectroscopy (^1^H NMR)

## Abstract

The covering oils of twenty-two commercially canned sardines were studied by Proton Nuclear Magnetic Resonance spectroscopy (^1^H NMR) freshly purchased and also after storage at room temperature for fifteen years. The filling oils studied were olive oils (one extra-virgin olive oil), sunflower oils, soybean oils, and vegetable oils (unspecified origin). The aim was to obtain qualitative and quantitative information on lipid composition, oxidative and hydrolytic status, and on the changes occurring during storage. Just after purchase, in all the samples, the migration of fish omega-3 polyunsaturated eicosapentaenoic (EPA, C20:5ω3) and docosahexaenoic (DHA, C22:6ω3) acyl groups was reported; the occurrence of oxidative or hydrolytic reactions was not observed. After storage, the main change in the spectra was the presence of signals due to hydrolytic compounds (mainly 1,3-diglycerides, together with 1,2-diglycerides, 1-monoglycerides, and lower proportions of 2-monoglycerides). In eleven samples very low concentrations of saturated aldehydes (alkanals) were detected, which is considered a low oxidative status. It is suggested that the above-mentioned partial glycerides and alkanals migrated from sardine muscle to the oils. The content in omega-3 lipids in the oils after storage indicated the occurrence of lipid interchange between the sardine muscle and the packing oil in both directions.

## 1. Introduction

Fish is known to provide high contents of important macro- and micronutrients for the human diet, such as omega-3 polyunsaturated lipids (ω3), especially eicosapentaenoic (EPA, C20:5ω3) and docosahexaenoic (DHA, C22:6ω3) acyl groups, essential aminoacids and proteins, liposoluble vitamins, and microelements, among others. In recent years, special attention has been paid to the potential of forage fish, as this affordable and abundant seafood category is considered as the most nutritious fish species with the lowest carbon footprint [1]. A species of forage fish widely consumed in some European countries are sardines, not only fresh, but also as canned products. Canning is applied to prolong the shelf life of this perishable food product. In this process, briefly, sardines are precooked, packed with a filling medium, sealed in a hermetic container, and subjected to thermal treatment (sterilization at 110–130 °C for 25–120 min) to achieve commercial sterility [2]. As a result of this severe heat treatment, fish enzymes and microorganisms are inactivated. Afterwards, the maturation process starts and continues during storage until the cans are consumed.

In canned sardines, the filling medium is generally a vegetable oil, but other kinds of covering medium are also possible, like tomato sauce or marinade [3]. The quality and nature of the oils employed are of great importance as they can affect not only the nutritional and sensory value of sardines but also their price and shelf life. It must be noted that although there are several studies on the nutritional and quality control of canned fish products, there are not so many on that of their covering oils [4,5,6,7]. Some studies focused on the assessment of the genuineness of the oil used as liquid medium, but this is a difficult task due to the lipid interchange that occurs between the covering oil and the fish [8]. Moreover, just three studies have focused on the presence of oxidative and hydrolytic compounds in the covering oils of canned fish [9,10,11], which is a subject of great interest due to its potential implications for consumers’ health. In this regard it must be noted that in recent years, special attention has been paid to the addition of potential natural antioxidants to the packing medium of canned sardines [12] and other fish species [13,14] to enhance lipid stability. Regarding the methodology employed to study lipid main components, in most of these previous works, derivatization of the fatty acyl chains to the corresponding methyl esters (FAMEs) followed by gas chromatographic (GC) methods was used. This is quite laborious, and involves a complex series of chemical manipulations that, together with the high temperatures of GC injector and oven, may give rise to the artifactual oxidation of the compounds subjects of study [15].

In recent decades, interest has emerged in the use of spectroscopic techniques, such as Proton Nuclear Magnetic Resonance spectroscopy (^1^H NMR), to study food lipid samples as a whole, as this technique can provide qualitative and quantitative information on lipid components and on the occurrence of degradative reactions, like oxidation and hydrolysis, in a single run of a few minutes and without any previous sample modification [16,17,18,19].

In this context, the aim of this work is to study, by ^1^H NMR, the quality and safety of the covering oils of commercial canned sardines, not only freshly purchased but also after being stored for fifteen years at room temperature. Special attention will be paid to the potential changes in the composition in main acyl groups of the packing oils, and also to the presence of oxidative or hydrolytic compounds. This is the first time that this kind of study has been carried out for such a long-term storage period.

## 2. Materials and Methods

### 2.1. Samples

Twenty-two commercially canned sardines (*Sardina pilchardus*, also called European sardine) of different brands were acquired in local supermarkets (four cans of each sample) [3]. These twenty-two samples were selected because they represented all the different canned sardines that were marketed at that time (2010) in local supermarkets in Vitoria-Gasteiz (Spain). According to the labelling, four kinds of oils were employed as covering mediums: olive oil (OO) in seven samples (OO5 sample contained extra-virgin olive oil, but the others were made of refined olive oil blended with virgin olive oil); sunflower oil (SFO) in six samples; soybean oil (SYO) in four samples; and vegetable oil of unspecified origin (VO) in five samples. It was only possible to find one sample of sardines canned in extra-virgin olive oil; probably due to its high price, this higher quality oil is used in more expensive canned fish, such as tuna [11]. It must be noted that in most of the samples, the covering medium was the oil, but in three of them, in addition to oil, they also contained many other ingredients like vinegar or vegetables; to point out this fact, the name of these samples was marked with an asterisk: SFO6*, SYO3*, and SYO4*. The information provided by the producers in the labelling is shown in Supplementary Table S1. Just after acquiring the cans in 2010, two cans of each sample were opened and the covering oil was separated by decantation, followed by filtration through anhydrous sodium sulphate (Panreac, Barcelona, Spain). Samples were frozen at −20 °C until their study by ^1^H NMR.

### 2.2. Storage

Remaining canned sardines were kept closed in a cupboard at room temperature in the dark for fifteen years. Afterwards, two cans of each sample were opened, and their covering oil was studied as described above. The samples were named similarly but with the prefix ‘ST_’.

### 2.3. ^1^H Nuclear Magnetic Resonance Spectra Acquisition and Derived Data

The ^1^H NMR spectra of the samples were acquired in duplicate using a Bruker Avance 400 spectrometer operating at 400 MHz (Bruker Scientific Instruments, Billerica, MA, USA). For sample preparation, 200 μL of oil was mixed in a 5 mm diameter ^1^H NMR tube with 400 μL of deuterated chloroform (CDCl_3_, 99.8%), containing a small proportion of non-deuterated chloroform and 0.03% of tetramethylsilane (TMS). This was used as reference compound to calibrate the chemical shift at 0.0 ppm. NMR deuterated solvent was acquired from Eurisotop (Saint-Aubin, France). The acquisition parameters were as follows: spectral width 6250 Hz, relaxation delay 3 s, number of scans 64, acquisition time 2.621 s, and pulse width 90°, with a total acquisition time of 6 min and 20 s. The assignment of the ^1^H NMR signals (based on their chemical shifts and multiplicities) was performed using standards and/or the literature, as previously described, and is provided in Table 1 [16,17,18,19,20,21]. The relaxation delay and acquisition time used to obtain the spectra ensured complete relaxation of the sample protons, allowing the signal area to be proportional to the number of protons generating them. This enabled the use of ^1^H NMR signals to estimate the molar percentage of the several kinds of acyl groups, glycerides, and oxidation products present in the samples, as described in the Supplementary Material. The ^1^H NMR spectra illustrating the two figures were plotted at a fixed absolute intensity value to ensure validity for comparative purposes and processed using the MNova program (Mestrelab Research, Santiago de Compostela, Spain).

### 2.4. Statistical Analysis

Data provided in Table 2, Table 3 and Table 4 are average values of the several determinations of each sample, together with the corresponding standard deviation, calculated using Microsoft Excel 2016.

## 3. Results and Discussion

### 3.1. ^1^H NMR Study of the Covering Oils of Canned Sardines Freshly Purchased

**Figure 1:**
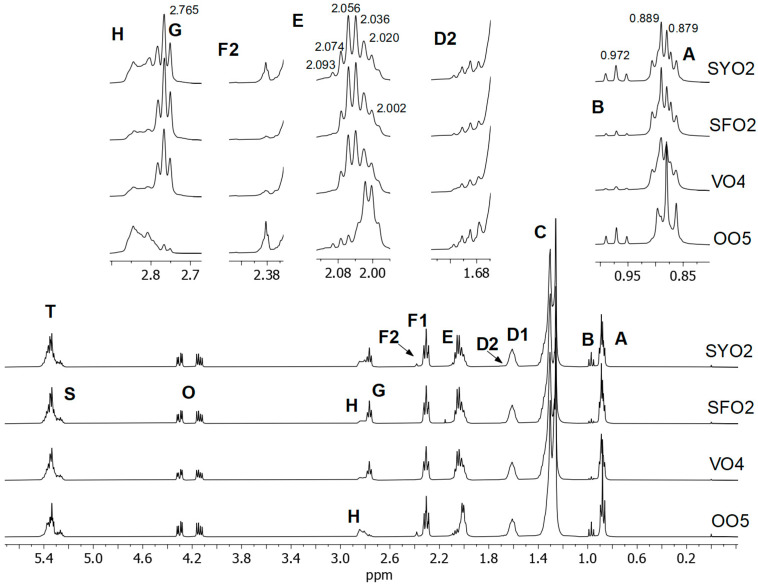
^1^H NMR spectra between 0 and 5.5 ppm of covering oils of different nature: soybean oil (SYO2), sunflower oil (SFO2), vegetable oil (VO4), and extra-virgin olive oil (OO5). Some spectral regions were properly enlarged in the upper part of the figure for comparative purposes. Signal letters agree with those in Table 1.

It is well known that vegetable oils, like fish lipids, are composed mainly of triglycerides (TG), which can have saturated, mono-, di-, and polyunsaturated acyl groups (AG) in different proportions depending on their nature. The protons of these acyl groups generate different main signals from 0 to 5.5 ppm in the ^1^H NMR spectra. Figure 1 shows the spectra of four filling oils of different nature; these are soybean (SYO2), sunflower (SFO2), vegetable oil of unspecified origin (VO4), and olive oil (OO5, extra-virgin olive oil). In the upper side of this figure, some signals have been properly enlarged for comparative purposes. The assignment of the signals is given in Table 1, in agreement with previous studies [16,17,18,19,20,21].

Signal **A**, between 0.84 and 0.94 ppm, is due to the overlapping of the triplets of methylic protons of the saturated (Sat), monounsaturated ω9 and/or ω7 (MU) acyl groups, and that of the diunsaturated ω6 (DUω6) acyl groups. Thus, signal A can provide six clearly distinguishable peaks, three of them belonging to the methylic protons of saturated and monounsaturated acyl groups (triplet centred at 0.879 ppm) and the other three belonging to diunsaturated acyl groups (triplet centred at 0.889 ppm). As can be observed in the spectra of SYO2, SFO2, and VO4, the triplet at 0.889 ppm is higher than the triplet at 0.879 ppm, evidencing a higher proportion of diunsaturated ω6 acyl groups (mainly linoleic, C18:2ω6) than of saturated and monounsaturated acyl groups. The opposite is observed in signal A of OO5 spectrum, in agreement with the high content of monounsaturated oleic acyl groups (C18:1ω9) in olive oils.

Signal **B**, between 0.94 and 1.00 ppm, is due to the triplet of methylic protons of ω3 polyunsaturated acyl groups, which is centered at 0.972 ppm and can correspond either to the linolenic acyl groups of the original vegetable oil (C18:3ω3), or, if migration has occurred, also to the ω3 sardine lipids; this is mainly EPA and DHA [22]. As can be observed, signal B shows a higher intensity in the spectra of SYO2 and OO5 than in those of SFO2 and VO4. This is in agreement with the high content in linolenic acyl groups in soybean oils (4.5–11.0%), and with their almost complete absence in sunflower oils (<0.3%) [23]. Regarding OO5 spectra, the intensity of signal B is much higher than that of a typical olive oil, which contains low proportions of linolenic (<1.5%); this fact suggests that in this covering oil, ꞷ3 fish lipids are also present.

Signal **C**, between 1.19 and 1.42 ppm, is due to methylenic protons either in position beta, or further in relation to double bonds, or in position gamma, or further in relation to the carbonyl group in the different acyl groups. It has two main peaks, one at 1.257 ppm corresponding to the methylenic protons of saturated acyl groups and the other one near 1.300 ppm associated with the overlapping of methylenic protons of all the unsaturated acyl groups [17]. In addition, it has a shoulder near 1.280 ppm related to monounsaturated acyl groups. This was observed in the OO5 spectrum, but it is not enlarged in Figure 1.

Signal **D1**, between 1.54 and 1.67 ppm, is due to methylenic protons in the beta position in relation to the carbonyl group, except those of EPA and DHA acyl groups (see Figure 1). As is known, this signal does not show significant differences in multiplicity or in chemical shifts between the ^1^H NMR spectra of the different acyl groups [17]. Signal **D2**, between 1.67 and 1.74 ppm, is due to methylenic protons in the beta position in relation to the carbonyl group of EPA acyl groups. Thus, although it is absent in the ^1^H NMR spectra of vegetable oils [17], it can be observed in the enlarged spectra of the covering oils shown in Figure 1, especially in OO5 and SYO2, evidencing the migration of EPA acyl groups from the sardine muscle to the packing oil.

Signal **E**, between 1.92 and 2.15 ppm, is due to the overlapping of the various signals of allylic protons, that is, of alpha-methylenic protons in relation to a single double bond in the different acyl groups, except those of DHA acyl group, which are also in beta-position in relation to the carbonyl group. As saturated acyl groups do not have double bonds, they make no contribution to this signal. Regarding this signal, the olive oil sample OO5 is clearly distinguishable from the other covering oils, because the peaks at 2.002 and 2.020 ppm corresponding to monounsaturated acyl groups are much higher than those at 2.036, 2.056, and 2.074 ppm due to diunsaturated ω6 acyl groups (mainly linoleic). It must be noted that in the spectra of OO5 and SYO2, a peak at 2.093 ppm can be observed due to the allylic protons of ω3 acyl groups. This is in agreement with what was observed in signal B.

Signal **F1**, between 2.26 and 2.36 ppm, is due to methylenic protons in the alpha-position in relation to the carbonyl group, except those of DHA acyl group (see Figure 1). This signal does not show significant differences in multiplicity or in chemical shifts between the ^1^H NMR spectra of the different acyl groups supported on triglycerides; for this reason, it is not useful to discriminate covering oils. Signal **F2** between 2.37 and 2.41 is due to methylenic protons in alpha and beta-positions in relation to the carbonyl group of DHA acyl groups, being present in the spectra of fish lipids but not in those of vegetable oils [16,20]. As shown in the enlarged spectra of Figure 1, signal F2 is present in the four covering oils, mainly in OO5 and SYO2; this is in agreement with the above commented on signal D2 due to EPA acyl groups.

Signal **G** is a triplet of the bis-allylic protons of diunsaturated ω6 (linoleic) acyl groups, that is of their methylenic protons in α-position in relation to two double bonds. This signal is partially overlapped with Signal **H**, also due to the bis-allylic protons but of the rest of polyunsaturated acyl groups, which are mainly ω3. As can be observed in the spectra of Figure 1, the intensity of signal G is much higher in SYO2, SFO2, and VO4 than in OO5, in agreement with that commented on above regarding signals A and E. On the other hand, the intensity of signal H is higher in OO5 and SYO2 than in SFO2 and VO4, which is in agreement with that reported concerning signals B, D2, E (peak at 2.093 ppm), and F2.

Furthermore, signals due to the protons of the glycerol backbone of TG can also be observed. Signal **O** at 4.10–4.34 ppm is due to the protons bonded to carbon atoms 1 and 3 of the glyceryl group, and signal **S** at 5.23–5.30 ppm due to those of carbon atom 2. The latter signal overlaps slightly with signal **T**, at 5.28–5.47 ppm, due to olefinic protons of all the unsaturated acyl groups. These signals O, S, and T do not show differences in multiplicity or chemical shifts between the different acyl groups and thus do not provide useful information for discriminating between different oil samples.

Not only qualitative but also quantitative information can be obtained from the study of these ^1^H NMR signals. Table 2 provides the molar percentages of the different kinds of acyl groups present in the filling oils. In general, olive oils showed the highest proportion of monounsaturated acyl groups (MU, mainly oleic) and the lowest of diunsaturated ω6 acyl groups (DUω6, mainly linoleic). In the seed oils, much higher proportions of DUω6 were observed. It is remarkable that in all the samples, the presence of DHA (0.8–8.3%) and of EPA (1.0–9.0%) was reported in variable proportions. These results are in agreement with previous studies carried out by chromatographic techniques that reported the interchange of lipids between canned sardine muscle and the olive oil used as packing medium [8,24], which suggested that there is a diffusion gradient that causes that the acyl group proportions of sardine lipids and the covering oil tend to be similar. In other fish species, like tuna canned in soybean oil, similar results have also been reported by chromatographic techniques [5]. As can be observed in Table 2, soybean oil samples contained the highest proportions of total ꞷ3 lipids, due to the fact that in addition to DHA and EPA migrated from sardine muscle, this kind of oil naturally contains higher proportions of linolenic acyl groups (4.5–11.0%) than olive (<1.5%) or sunflower (<0.3%) oils [23]. Regarding saturated acyl groups, variable proportions were detected in all the samples. The highest proportion of saturated acyl groups was observed in a soybean oil sample, SYO4*. As this sample also contained the highest proportions of DHA and EPA, this fact could be attributed to the migration of sardine saturated lipids to the filling oil, in agreement with previous studies on sardines canned in olive oil [24]. It must be noted that this sample SYO4* is one of those in which the oil is not the main ingredient of the filling medium, and thus fish lipids are more concentrated than in other samples (see Table S1). The fact that these are commercial samples prepared from raw materials of unknown initial composition, which have different final weights and best-before dates (see Table S1) and which have undergone different manufacturing processes, makes it difficult to draw further conclusions about the variability of the proportions of acyl groups in the covering oils reported in Table 2.

In short, ^1^H NMR provided a great deal of information on the lipid composition of the covering oils. It allowed us to detect the migration of sardine ω3 polyunsaturated lipids, evidenced by the presence in the spectra of signal D2 related to EPA and signal F2 related to DHA acyl groups, which are absent in the typical spectra of vegetable oils. Moreover, signals A and E made it possible to distinguish with the naked eye the ^1^H NMR spectra of olive oils from the other seed oil samples, due to the high content of monounsaturated oleic acyl groups in the former. However, the spectra of sunflower, soybean, and vegetable oils showed many similarities, probably because most of the oils labelled as ‘vegetable’ are, indeed, sunflower oil, soybean oil, or mixtures thereof, among others. This fact, together with the migration of fish lipids, makes the assessment of the genuineness of the oil used as liquid medium in canned sardines difficult. Similar results were reported by means of attenuated total reflection Fourier transform infrared spectroscopy (ATR-FTIR) and chemometrics on the authentication of packing oils from commercial canned tuna, which differentiated olive oil from seed oils (sunflower and vegetable oils) [25]. In this regard, it must be noted that if a huge number of ^1^H NMR spectra of covering oils of different nature were studied together with the power of multivariate analyses, this technique could be a very good fingerprinting and discriminating method [26].

As for the oxidative status of the covering oils, no proton signals related to primary or to secondary oxidation products were observed in their ^1^H NMR spectra [18], which evidenced that no relevant lipid oxidation reactions had occurred in the covering oils. These results are in agreement with those obtained in the study of the oxidative status of covering olive oils of canned tuna by Peroxide Value [9]. On the contrary, a higher oxidative degradation level was reported in seed oils (sunflower, soybean, corn, vegetable) than in olive oils used as filling mediums in canned sardines, tuna, anchovies, and mackerel, according to their percentage of polar compounds [10,11].

Concerning the hydrolytic status of the covering oils, in the spectra of all the samples, incipient signal **J** at 3.73 ppm was observed; this is due to the methylenic protons in carbon atom 3 of the glyceryl backbone of 1,2-diglycerides (1,2-DG). This small proportion of 1,2-DG is typically present in vegetable oils and is considered negligible. In a previous study, a higher hydrolytic level (Free Fatty Acids percentage, FFA%) was reported for olive oils than for seed oils used as filling mediums in different canned fish, but this fact was attributed to the refining process of seed oils that remove these compounds. Regardless, in all the samples, FFA% was below the legal limits established for the different types of oils [10,11].

### 3.2. ^1^H NMR Study of the Covering Oils of Canned Sardines After Fifteen-Year Storage

**Figure 2:**
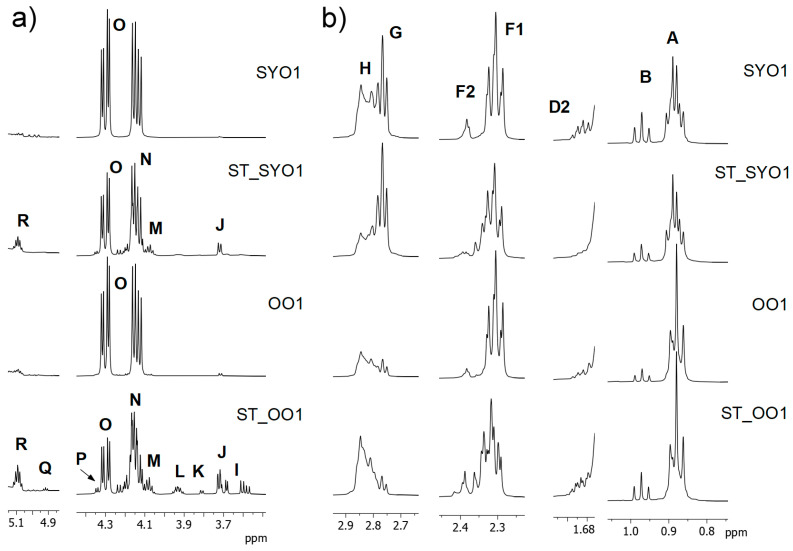
Some ^1^H NMR spectral regions of two samples before (SYO1, OO1) and after being submitted to storage at room temperature for fifteen years (ST_SYO1, ST_OO1): (**a**) Regions where protons in the glyceryl backbone of mono-, di-, and triglycerides are observed, (**b**) Regions where the main signals related to polyunsaturated omega-3 lipids are observed. Signal letters agree with those in Table 1.

When the ^1^H NMR spectra of the covering oils submitted to fifteen-year storage were studied, the main change observed was the appearance of new signals between 3.6 and 5.1 ppm due to protons in the glyceryl backbone of mono- and diglycerides. Figure 2a shows these enlarged regions of the spectra of one soybean and one olive covering oils before (SYO1, OO1) and after storage (ST_SYO1, ST_OO1) for comparative purposes. In addition to signal O due to protons in the glyceryl backbone of TG, which is typical in the spectrum of edible oils, new signals can be observed due to protons in the glyceryl backbone of partial glycerides (see Table 1): signal **M** to 1,3-diglycerides (1,3-DG), signals **J**, **P** and **R** related to 1,2-diglycerides (1,2-DG), signals **I**, **L** and **N** to 1-monoglycerides (1-MG), and signals **K** and **Q** to 2-monoglycerides (2-MG).

Table 3 shows the average molar percentages of the TG, 1,3-DG, 1,2-DG, 1-MG, 2-MG, and glycerol (Gol) detected in the covering oils after storage. It must be noted that in edible oils, TG accounts for approximately 97–98% of total glycerides present. As can be observed in Table 3, in stored covering oils, TG ranged from 78.7% in ST_OO5 to 37.9% in ST_OO1. The spectrum of the latter sample is shown in Figure 2a and a great decrease in the intensity of signal O can be observed after storage. The partial glyceride that showed the highest proportions in the covering oils were 1,3-DG, ranging from 24.0% in ST_OO1 to 12.0% in ST_OO5. They were generated due to the breakdown of the ester bond in carbon 2 of the glyceryl backbone of TG, also releasing one fatty acid (FA). Moreover, 1,2-DG were also detected in the samples, but in lower proportions (ranging from 9.9% in ST_OO1 to 5.0% in ST_OO2 and ST_OO5). They were formed due to the breakdown of the ester bond in carbon 3 of the glyceryl backbone of TG. Regarding monoglycerides, 1-MG were present in all samples, with slightly lower proportions than those of 1,2-DG (ranging from 15.6% in ST_OO1 to 3.0% in ST_OO5). On the other hand, 2-MG were also detected in all samples, but in much lower proportions (<1.0%), which were lower than those of Gol. The higher proportions of 1-MG than 2-MG can be explained by the above-mentioned preferential formation of 1,3-DG, because as hydrolysis advances, 1,3-DG give rise to 1-MG and one FA. These results are in agreement with previous studies carried out by ^13^C NMR on lipid hydrolysis occurring in raw and heat-treated tuna muscle during industrial canning [27,28]. During frozen storage of raw muscle, a preferential formation of 1,2-DG was reported due to the activity of lipolytic enzymes. However, after thermal processing (cooking and sterilization in cans), a different lypolisis mechanism was evidenced, reporting a preferential formation of 1,3-DG. These results were attributed to a physical breakdown of the ester bond in carbon atom 2 of TG due to a heat effect, because lipase enzymes were not active due to the high temperatures applied during canning [27].

This increase in partial glycerides observed in all the covering oils after storage is in agreement with other studies on canned tuna stored for up to 6 years, which reported that an increase in the storage time produced higher FFA% in fish muscle and in the covering oil (olive oil) [9]. In that study, cans that contained only olive oil (blank oils without tuna) were also submitted to the same processing conditions, and after long-term storage no increase of FFA% was observed. Therefore, the above-mentioned increase of FFA% in the covering oils was attributed to their migration from tuna muscle.

Regarding the main lipid components in the stored covering oils, due to the presence of partial glycerides and FA, in addition to the initial TG and AG, as can be observed in Figure 2b, some changes were observed in multiplicity and chemical shifts in the ^1^H NMR signals generated by the protons supported on alpha and beta carbon atoms in relation to the carbonyl/carboxyl groups of glycerides and FA (signals D1, D2, F1, and F2) due to small differences in the chemical environment [19]. Because of this, it was only possible to estimate the molar percentage of total ꞷ3 AG and FA in the stored covering oils. As shown in Table 3, different changes occurred in the ꞷ3% of the samples after storage. In most of the samples, the proportion increased after storage; in one of them it remained similar (OO7), but in five of them it decreased (SFO4, SYO1, SYO2, SYO4, VO3). As an example, in the spectra of Figure 2b, it can be observed that after storage, the intensity of signal B due to methylic protons of ω3 polyunsaturated lipids increased in ST_OO1 sample and decreased in ST_SYO1. These results are in agreement with previous studies during shorter periods of storage (4 months and 5 years), which reported that during storage of canned sardines, a lipid interchange between sardines and the covering oil occurred in both directions, with fish being able to reabsorb the lipids previously released during sterilization [8,24].

Finally, concerning the oxidative status of the covering oils after long-term storage, in their ^1^H NMR spectra, no proton signals related to primary oxidation products were observed. Nevertheless, it must be noted that in eleven samples, an incipient signal at 9.75 ppm due to the aldehydic proton of alkanals was observed. As Table 4 shows, the estimated concentration in the samples was up to 0.1 mmol per total number of moles of AG and FA, which is a low value, similar to that reported previously in just acquired sunflower oils [29]. It is remarkable that unsaturated aldehydes were not detected, because in previous studies by ^1^H NMR on sunflower oils stored in closed receptacles at room temperature for up to 10 years, saturated and unsaturated aldehydes, like *trans*-2-alkenals and *trans*,*trans*-2,4-alkadienals, were simultaneously detected [30]. Therefore, it is suggested that the alkanals present in the covering oils migrated from the sardine muscle during storage, in agreement with the above-commented on hydrolytic products. This low oxidative status of the covering oils after long-term storage of canned sardines can be explained by the low oxygen availability in the hermetically sealed cans and also by the storage conditions (room temperature in the dark). These results are in agreement with the lack of oxidative development observed in fish muscle and in the covering oil (olive) of canned tuna stored for up to 6 years, determined by Conjugated Dienes content [9].

## 4. Conclusions

The qualitative and quantitative study of the ^1^H NMR spectra of the covering oils of canned sardines freshly purchased and after long-term storage provided a great deal of information not only on the changes occurring in their lipid composition, but also on the presence of oxidative and hydrolytic compounds. Just after purchase, in all the samples, the migration of ꞷ3 EPA and DHA acyl groups from the sardine muscle to the packing oils was observed, whereas no oxidative or hydrolytic compounds were detected. Taking these results into account, the consumption of the covering oils of tinned sardines can be considered of interest from a nutritional point of view, especially if high quality vegetable oils are employed, like extra-virgin olive oil or olive oil.

After long-term storage for fifteen years, the main change reported in the filling oils was the presence of lipolytic products, evidenced by the detection of 1,3-diglycerides, 1,2-diglycerides, 1-monoglycerides, and lower proportions of 2-monoglycerides. It must be highlighted that 1,3-diglycerides were the most abundant partial glycerides present in all samples. Regarding the oxidative status, in eleven samples, very low concentrations of saturated aldehydes (alkanals) were observed, similar to those reported previously in other just acquired vegetable oils. Taking into account previous studies, it is suggested that the above-mentioned partial glycerides and alkanals detected in the covering oils after storage migrated from sardine muscle to the oils. In addition, the content in total ꞷ3 lipids increased in most of the samples after storage, but decreased in five of them, evidencing that lipid interchange between the sardine muscle and the covering oil occurred in both directions during storage, which can be of interest from the nutritional point of view. It can be concluded that, considering all the changes that have been reported, during the course of long-term storage of canned sardines, the quality and safety of the covering oils were scarcely affected. At this point, it must be noted that canned sardines have a “best-before date”, not an “expiration date”. The best-before date indicates until what date the food keeps its properties intact, as long as the hermetic package is closed. After this date (some years), food organoleptic properties start to change, but from the microbiological point of view it is still safe to consume, as long as the can is intact and has no dents or dings. This latter could indicate that the can contains *Clostridium botulinum* toxin, and thus it should be discarded immediately.

## Figures and Tables

**Table 1:** Chemical shift assignments and multiplicities of the ^1^H NMR signals in CDCl_3_ of the main protons of glycerides and fatty acids, and of certain oxidation compounds present in the covering oils of canned sardines before and after fifteen-year storage, in agreement with previous studies [16,17,18,19,20,21]. The signal letters agree with those given in Figure 1 and Figure 2.

Signal	Chemical Shift (ppm)	Multi-Plicity	Functional Group	
			Type of Protons	Compound
	Main Acyl Groups (AG) and Fatty Acids (FA)			
**A**	0.88	t	-C**H_3_**	saturated, monounsaturated **ω-9** and/or **ω-7 AG** and **FA**
	0.89	t	-C**H_3_**	unsaturated **ω-6 AG** and **FA**
**B**	0.97	t	-C**H_3_**	unsaturated **ω-3 AG** and **FA**
**C**	1.19–1.42	m *	-(C**H_2_**)_n_-	**AG** and **FA**
**D1**	1.61	m	-OCO-CH_2_-C**H_2_**-	**AG** in **TG**, except for DHA and EPA AG
	1.62	m	-OCO-CH_2_-C**H_2_**-	**AG** in **1,2-DG**, except for DHA and EPA AG
	1.63	m	-OCO-CH_2_-C**H_2_**-, COOH-CH_2_-C**H_2_**-	**AG** in **1,3-DG**, **1-MG** and **FA**, except for DHA and EPA AG/FA
	1.64	m	-OCO-CH_2_-C**H_2_**-	**AG** in **2-MG**, except for DHA and EPA AG
**D2**	1.69	m	-OCO-CH_2_-C**H_2_**-	EPA **AG** in **TG**
	1.72	m	COOH-CH_2_-C**H_2_**-	EPA **FA**
**E**	1.92–2.15	m **	-C**H_2_**-CH=CH-	**AG** and **FA**, except for -CH_2_- of DHA AG/FA in β-position in relation to carbonyl group
**F1**	2.26–2.36	dt	-OCO-C**H_2_**-	**AG** in **TG**, except for DHA AG
	2.33	m	-OCO-C**H_2_**-	**AG** in **1,2-DG**, except for DHA AG
	2.35	t	-OCO-C**H_2_**- COOH-C**H_2_**-	**AG** in **1,3-DG**, **1-MG** and **FA**, except for DHA AG/FA
	2.38	t	-OCO-C**H_2_**-	**AG** in **2-MG**, except for DHA AG
**F2**	2.37–2.41	m	-OCO-C**H_2_**-C**H_2_**-	DHA **AG** in **TG**
	2.39–2.44	m	COOH-C**H_2_**-C**H_2_**-	DHA **FA**
**G**	2.77	t	=HC-C**H_2_**-CH=	**diunsaturated ω-6 AG** and **FA**
**H**	2.77–2.90	m	=HC-C**H_2_**-CH=	**polyunsaturated ω-3** (and ω-6) **AG** and **FA**
**I**	3.65	ddd	ROCH_2_-CHOH-C**H_2_**OH	glyceryl group in **1-MG**
**J**	3.73	m ***	ROCH_2_-CH(OR′)-C**H_2_**OH	glyceryl group in **1,2-DG**
**K**	3.84	m ***	HOC**H_2_**-CH(OR)-C**H_2_**OH	glyceryl group in **2-MG**
**L**	3.94	m	ROCH_2_-C**H**OH-CH_2_OH	glyceryl group in **1-MG**
**M**	4.05–4.21	m	ROC**H_2_**-C**H**OH-C**H_2_**OR′	glyceryl group in **1,3-DG**
**N**	4.18	ddd	ROC**H_2_**-CHOH-CH_2_OH	glyceryl group in **1-MG**
**O**	4.22	dd,dd	ROC**H_2_**-CH(OR′)-C**H_2_**OR″	glyceryl group in **TG**
**P**	4.28	ddd	ROC**H_2_**-CH(OR′)-CH_2_OH	glyceryl group in **1,2-DG**
**Q**	4.93	m	HOCH_2_-C**H**(OR)-CH_2_OH	glyceryl group in **2-MG**
**R**	5.08	m	ROCH_2_-C**H**(OR′)-CH_2_OH	glyceryl group in **1,2-DG**
**S**	5.27	m	ROCH_2_-C**H**(OR′)-CH_2_OR′	glyceryl group in **TG**
**T**	5.28–5.7	m	-C**H**=C**H**-	**AG** and **FA**
	**Secondary oxidation compounds**			
	***Aldehydes***			
**a**	9.75	t	-C**H**O	alkanals

Abbreviations: t, triplet; m, multiplet; d, doublet; s, singlet; DHA, docosahexaenoate (C22:6ꞷ3); EPA, eicosapentaenoate (C20:5ω3); 1,3-DG, 1,3-diglyceride; 1-MG, 1-monoglyceride; TG, triglyceride; 1,2-DG, 1,2-diglyceride; 2-MG, 2-monoglyceride. * overlapping of multiplets of methylenic protons in the different acyl groups either in beta-position, or further, in relation to double bonds, or in gamma-position, or further, in relation to the carbonyl group; ** overlapping of multiplets of the alpha-methylenic protons in relation to a single double bond of the different unsaturated acyl groups; *** this signal shows different multiplicity if the spectrum is acquired from the pure compound or taking part in the mixture.

**Table 2:** Molar percentages of the main acyl groups of the covering oils of canned sardines freshly purchased in local supermarkets, estimated by ^1^H NMR.

Sample	Total ω3	DHA	EPA	DUꞷ6	MU	Total U	Sat
OO1	6.8 ± 0.0	2.8 ± 0.0	3.8 ± 0.0	7.6 ± 0.1	68.9 ± 0.9	83.2 ± 1.0	16.8 ± 1.0
OO2	5.7 ± 0.1	2.3 ± 0.1	3.3 ± 0.2	6.5 ± 0.2	73.4 ± 0.1	85.5 ± 0.1	14.5 ± 0.2
OO3	8.0 ± 0.1	3.7 ± 0.1	3.7 ± 0.1	8.3 ± 0.2	65.0 ± 0.6	81.3 ± 0.3	18.7 ± 0.3
OO4	4.0 ± 0.0	1.8 ± 0.0	1.7 ± 0.2	5.9 ± 0.2	75.4 ± 0.2	85.3 ± 0.1	14.7 ± 0.1
OO5	10.6 ± 0.1	4.0 ± 0.1	5.9 ± 0.2	6.4 ± 0.1	65.4 ± 0.4	82.4 ± 0.3	17.6 ± 0.3
OO6	7.9 ± 0.1	2.1 ± 0.0	5.7 ± 0.1	7.9 ± 0.0	69.9 ± 0.5	85.6 ± 0.6	14.4 ± 0.6
OO7	2.7 ± 0.4	0.9 ± 0.0	1.8 ± 0.2	6.7 ± 0.1	78.2 ± 1.0	87.6 ± 0.5	12.4 ± 0.5
SFO1	2.5 ± 0.1	0.8 ± 0.1	1.4 ± 0.1	55.1 ± 0.8	30.4 ± 0.8	88.0 ± 0.0	12.0 ± 0.0
SFO2	4.8 ± 0.1	0.9 ± 0.0	3.8 ± 0.2	54.4 ± 0.5	27.6 ± 1.4	86.7 ± 1.0	13.3 ± 1.0
SFO3	1.9 ± 0.1	0.8 ± 0.0	1.0 ± 0.1	64.0 ± 0.9	23.0 ± 1.4	88.9 ± 0.4	11.1 ± 0.5
SFO4	6.2 ± 0.1	2.1 ± 0.0	3.8 ± 0.0	45.9 ± 3.6	34.1 ± 3.7	86.2 ± 0.0	13.8 ± 0.0
SFO5	1.7 ± 0.1	0.6 ± 0.1	1.1 ± 0.2	66.4 ± 1.1	23.1 ± 0.2	91.2 ± 1.0	8.8 ± 1.0
SFO6*	13.3 ± 0.2	5.8 ± 0.2	6.5 ± 0.3	37.7 ± 1.3	31.5 ± 1.8	82.4 ± 0.4	17.6 ± 0.4
SYO1	15.2 ± 0.0	4.9 ± 0.1	6.0 ± 0.2	40.8 ± 2.6	24.0 ± 3.1	80.0 ± 0.5	20.0 ± 0.5
SYO2	12.9 ± 0.3	3.0 ± 0.1	5.6 ± 0.3	45.7 ± 0.5	23.7 ± 0.3	82.3 ± 0.0	17.8 ± 0.0
SYO3*	14.1 ± 0.1	3.4 ± 0.0	5.9 ± 0.1	41.9 ± 0.4	24.6 ± 0.4	80.6 ± 0.1	19.4 ± 0.1
SYO4*	21.5 ± 0.2	8.3 ± 0.1	9.0 ± 0.1	28.3 ± 0.5	25.9 ± 0.1	75.7 ± 0.6	24.3 ± 0.7
VO1	9.0 ± 0.3	1.7 ± 0.1	2.5 ± 0.1	51.5 ± 1.8	23.3 ± 2.5	83.8 ± 0.3	16.2 ± 0.4
VO2	2.1 ± 0.0	0.8 ± 0.0	1.2 ± 0.1	61.8 ± 1.8	25.0 ± 1.4	88.8 ± 0.4	11.2 ± 0.4
VO3	9.2 ± 0.0	4.4 ± 0.0	4.3 ± 0.0	32.2 ± 1.7	42.3 ± 0.3	83.8 ± 2.0	16.3 ± 2.0
VO4	4.8 ± 0.2	1.3 ± 0.3	3.2 ± 0.7	48.8 ± 2.3	32.8 ± 2.7	86.4 ± 0.2	13.6 ± 0.3
VO5	5.1 ± 1.0	2.3 ± 0.1	2.6 ± 1.0	55.3 ± 4.1	27.5 ± 4.6	87.9 ± 0.4	12.1 ± 0.4

OO: olive oil; SFO: sunflower oil; SYO: soybean oil; VO: vegetable oil; ω-3: omega-3; DHA: docosahexaenoic; EPA: eicosapentaenoic; DUꞷ6: diunsaturated omega-6; MU: monounsaturated, Sat: saturated; U: total unsaturated. Asterisked samples did not contain oil as the main ingredient of the filling medium.

**Table 3:** Molar percentages of the several glycerides, together with that of total ꞷ3 acyl groups (AG) or fatty acids (FA), estimated by ^1^H NMR, in the covering oils of canned sardines after being stored for fifteen years.

Sample	TG	1,3-DG	1,2-DG	1-MG	Gol	2-MG	Total ω3
ST_OO1	37.9 ± 0.2	24.0 ± 0.2	9.9 ± 0.1	15.6 ± 0.1	11.6 ± 0.6	1.0 ± 0.0	11.6 ± 0.1
ST_OO2	74.9 ± 0.5	13.6 ± 0.5	5.0 ± 0.2	4.8 ± 0.2	1.3 ± 0.2	0.4 ± 0.1	9.9 ± 0.0
ST_OO3	62.9 ± 0.1	19.7 ± 0.1	7.8 ± 0.2	7.0 ± 0.1	2.1 ± 0.1	0.5 ± 0.0	10.7 ± 0.1
ST_OO4	76.7 ± 0.9	13.5 ± 0.8	5.0 ± 0.8	3.6 ± 0.7	1.0 ± 0.1	0.3 ± 0.1	6.6 ± 0.1
ST_OO5	78.7 ± 0.4	12.0 ± 0.2	5.0 ± 0.2	3.0 ± 0.0	1.0 ± 0.0	0.3 ± 0.0	17.2 ± 0.1
ST_OO6	57.4 ± 0.4	21.2 ± 0.1	9.5 ± 0.1	8.6 ± 0.0	2.7 ± 0.6	0.6 ± 0.0	11.0 ± 0.1
ST_OO7	63.8 ± 0.5	19.5 ± 0.0	8.0 ± 0.0	6.5 ± 0.1	1.7 ± 0.4	0.5 ± 0.0	2.7 ± 0.1
ST_SFO1	59.0 ± 0.1	20.3 ± 0.1	9.8 ± 0.3	9.2 ± 0.2	1.0 ± 0.0	0.7 ± 0.0	4.4 ± 0.1
ST_SFO2	75.9 ± 0.3	14.1 ± 0.0	5.2 ± 0.0	3.7 ± 0.1	1.0 ± 0.2	0.2 ± 0.0	5.2 ± 0.1
ST_SFO3	62.4 ± 3.0	19.2 ± 0.6	8.8 ± 1.3	7.3 ± 0.7	1.8 ± 1.8	0.5 ± 0.1	3.2 ± 0.2
ST_SFO4	64.7 ± 0.1	18.8 ± 0.1	8.5 ± 0.0	7.0 ± 0.0	0.5 ± 0.2	0.6 ± 0.0	5.8 ± 0.1
ST_SFO5	62.0 ± 0.8	18.7 ± 0.7	9.3 ± 0.2	8.2 ± 0.1	1.2 ± 0.2	0.6 ± 0.1	2.6 ± 0.0
ST_SFO6*	75.6 ± 0.1	14.4 ± 0.1	5.5 ± 0.0	3.9 ± 0.0	0.3 ± 0.2	0.3 ± 0.0	15.3 ± 0.0
ST_SYO1	66.9 ± 0.0	18.4 ± 0.0	7.4 ± 0.1	5.7 ± 0.0	1.2 ± 0.0	0.5 ± 0.0	10.7 ± 0.0
ST_SYO2	56.2 ± 0.1	21.2 ± 0.0	9.3 ± 0.0	9.0 ± 0.0	3.8 ± 0.0	0.7 ± 0.0	11.5 ± 0.0
ST_SYO3*	51.6 ± 0.1	20.7 ± 2.9	9.5 ± 0.0	11.2 ± 0.0	6.1 ± 3.1	0.9 ± 0.0	25.3 ± 0.0
ST_SYO4*	69.8 ± 0.3	16.2 ± 0.0	7.6 ± 0.0	5.6 ± 0.0	0.4 ± 0.3	0.4 ± 0.0	19.0 ± 0.0
ST_VO1	53.9 ± 0.1	21.4 ± 0.0	9.5 ± 0.0	9.2 ± 0.0	5.4 ± 0.0	0.6 ± 0.0	12.5 ± 0.1
ST_VO2	53.7 ± 0.1	21.3 ± 0.1	9.9 ± 0.3	10.2 ± 0.0	4.2 ± 0.3	0.7 ± 0.0	3.4 ± 0.0
ST_VO3	75.4 ± 0.1	12.5 ± 0.2	5.8 ± 0.4	4.0 ± 0.2	2.0 ± 0.1	0.3 ± 0.0	8.5 ± 0.2
ST_VO4	59.0 ± 0.3	20.8 ± 0.1	8.6 ± 0.4	7.8 ± 0.0	3.3 ± 0.1	0.5 ± 0.0	6.4 ± 0.0
ST_VO5	61.4 ± 0.4	19.7 ± 0.1	9.0 ± 0.4	7.7 ± 0.1	1.4 ± 0.0	0.7 ± 0.1	9.2 ± 0.0

ST_OO: olive oil; ST_SFO: sunflower oil; ST_SYO: soybean oil; ST_VO: vegetable oil; TG: triglycerides; 1,3-DG: 1,3-diglycerides; 1,2-DG: 1,2-diglycerides; 1-MG: 1-monoglycerides; 2-MG: 2-monoglycerides; Gol: glycerol; ω-3: omega-3. Asterisked samples did not contain oil as the main ingredient of the filling medium.

**Table 4:** Concentration of alkanals expressed as millimol per total number of moles of acyl groups (AG) and fatty acids (FA), estimated by ^1^H NMR in the covering oils of canned sardines before and after being stored for fifteen years.

Samples	Alkanals	Samples	Alkanals
OO1	-	ST_OO1	-
OO2	-	ST_OO2	0.1 ± 0.1
OO3	-	ST_OO3	-
OO4	-	ST_OO4	0.1 ± 0.0
OO5	-	ST_OO5	-
OO6	-	ST_OO6	0.1 ± 0.0
OO7	-	ST_OO7	0.1 ± 0.0
SFO1	-	ST_SFO1	-
SFO2	-	ST_SFO2	0.1 ± 0.0
SFO3	-	ST_SFO3	0.1 ± 0.0
SFO4	-	ST_SFO4	0.1 ± 0.0
SFO5	-	ST_SFO5	-
SFO6*	-	ST_SFO6*	0.1 ± 0.0
SYO1	-	ST_SYO1	-
SYO2	-	ST_SYO2	-
SYO3*	-	ST_SYO3*	-
SYO4*	-	ST_SYO4*	0.1 ± 0.0
VO1	-	ST_VO1	-
VO2	-	ST_VO2	-
VO3	-	ST_VO3	0.1 ± 0.0
VO4	-	ST_VO4	0.1 ± 0.0
VO5	-	ST_VO5	-

OO: olive oil; SFO: sunflower oil; SYO: soybean oil; VO: vegetable oil; * Asterisked samples did not contain oil as the main ingredient of the filling medium.

## Data Availability

The original contributions presented in the study are included in the article, further inquiries can be directed to the author.

## Supplementary Materials

The following supporting information can be downloaded at: https://www.mdpi.com/article/10.3390/foods14091589/s1, Table S1: Oil samples subject of study together with some information provided by the producer in the labelling of the canned sardines: denomination of each product, list of ingredients, best-before date, net weight and drained weight; Quantification from ^1^H NMR spectral data.
