# Supplementary material for: 1H NMR Study of the Lipid Composition, Oxidative and Hydrolytic Status of the Covering Oils of Canned Sardines After Long-Term Storage
Source: Foods. 2025 Apr 30;14(9):1589. doi: 10.3390/foods14091589 (PMC12071350; doi:10.3390/foods14091589)
Supplement: Supplementary file 1 [file foods-14-01589-s001.zip › foods-3589623-supplementary.pdf]

^{1}H NMR study of the lipid composition, oxidative and hydrolytic status of the covering oils of canned sardines after long-term storage

Encarnacion GOICOECHEA-OSES*

Food Technology, Faculty of Pharmacy, Lascaray Research Center, University of the Basque Country (UPV/EHU), Vitoria-Gasteiz, Spain.

*Corresponding author: encarnacion.goicoechea@ehu.eus

Table S1. Oil samples subject of study together with some information provided by the producer in the labelling of the canned sardines: denomination of each product, list of ingredients, best-before date, net weight and drained weight.

Oil sample	Denomination of the product in the label	Ingredients	Best before date (year)	Net weight (g)	Drained weight (g)
**OLIVE OIL**					
**OO1**	Small sardines in olive oil	Sardines (*Sardina pilchardus*), olive oil (27%) and salt	2015	81	57
**OO2**	Small sardines in olive oil	Sardine (*Sardina pilchardus*), olive oil and salt	2015	83	63
**OO3**	Small sardines in olive oil	Sardines, olive oil and salt	2015	90	65
**OO4**	Small sardines in olive oil (low-salt)	Sardines and olive oil	2014	90	65
**OO5**	Sardines in extra-virgin olive oil	Sardines (*Sardina pilchardus*), extra-virgin olive oil and salt	2014	120	85
**OO6**	Small sardines with lemon in olive oil	Sardines (*Sardina pilchardus*), refined olive oil, lemon slice and salt	2015	88	62
**OO7**	Small sardines with *Padrón* pepper in olive oil	Small sardines, olive oil, *Padrón* pepper and salt	2015	120	88
**SUNFLOWER OIL**					
**SFO1**	Small sardines in sunflower oil	Small sardines, sunflower oil and salt	2013	81	57
**SFO2**	Sardines in sunflower oil	Sardines (*Sardina pilchardus*), sunflower oil and salt	2015	115	8
**SFO3**	Small sardines with lemon in sunflower oil	Sardine (*Sardina pilchardus*), sunflower oil (26.5%), lemon slice (2%) and salt	2014	81	57
**SFO4**	Smoked small sardines in sunflower oil	Sardine (*Sardina pilchardus*), sunflower oil (28%), salt and smoke aroma	2014	81	57
**SFO5**	Small sardines in spicy sauce with sunflower oil	Sardine (*Sardina pilchardus*), sunflower oil (27%), salt and aroma	2013	81	57
**SFO6***	**Spicy sardines**	Sardines (*Sardina pilchardus*), sunflower oil, tomato, red pepper, carrot, pickle, onion, spices, natural aroma and salt	2014	120	85
**SOYBEAN OIL**					
**SYO1**	Small sardines in soybean oil	Sardines, soybean oil and salt	2014	90	65
**SYO2**	Spicy small sardines in soybean oil	Sardines, soybean oil, spices and salt	2015	90	65
**SYO3***	Sardines marinated **in vinegar**	Sardines, soybean oil, vinegar, spices and salt	2013	120	88
**SYO4***	Sardines **in tomato sauce**	Sardines, soybean oil, tomato, onion, spices and salt	2013	120	88
**VEGETABLE OIL**					
**VO1**	Small sardines in vegetable oil	Sardines, vegetable oil and salt	2014	85	60
**VO2**	Spicy small sardines in vegetable oil	Sardines, vegetable oil, spices and salt	2015	85	60

**VO3**	Smoked small sardines in vegetable oil	Sardines, vegetable oil, smoke aroma and salt	2014	90	65
**VO4**	Small sardines with lemon aroma in vegetable oil	Sardines (small sardines), vegetable oil, natural lemon aroma and salt	2013	81	57
**VO5**	Small sardines with lemon in vegetable oil	Sardines, vegetable oil, lemon slice and salt	2014	90	65

* Asterisked samples did not contain oil as the main ingredient of the filling medium.

1. Quantification from ^{1}H NMR spectral data

1.1. Main acyl groups in lipid samples before storage

The following equations were used to obtain the molar percentage of the main acyl groups present in the lipids of the covering medium of canned sardines before storage: total omega-3 acyl groups (ω3%), saturated plus monounsaturated and diunsaturated acyl groups (SMDU%), docosahexaenoic acyl groups (DHA%), eicosapentaenoic acyl groups (EPA%), total unsaturated acyl groups (U%), diunsaturated omega-6 acyl groups, mainly linoleic (DUω6%), saturated acyl groups (Sat%) and the rest of unsaturated acyl groups, which are mainly monounsaturated omega-9 (oleic) and omega-7, although other minor unsaturated acyl groups would also be included in this parameter, if present (MU%):

$$\omega3\% = 100A_B/(A_A+A_B) \quad \text{[eq.S1]}$$

$$SMDU\% = 100A_A/(A_A+A_B) \quad \text{[eq.S2]}$$

$$DHA\% = 100\, A_{F2}/(A_{F2}+2A_{F1}) \quad \text{[eq.S3]}$$

$$EPA\% = 100(2A_{D2})/(A_{F2}+2A_{D1}+2A_{D2}) \quad \text{[eq.S4]}$$

$$U\% = 100(2A_E+A_{F2})/(6A_O) \quad \text{[eq.S5]}$$

$$DU\omega6\% = 100(2A_G/3A_O) \quad \text{[eq.S6]}$$

$$Sat\% = 100 – U\% \quad \text{[eq.S7]}$$

$$MU\% = SMDU\% - DU\omega\text{-}6\% - S\% \quad \text{[eq.S8]}$$

where A_B, A_A, A_{F2}, A_{F1}, A_{D2}, A_{D1}, A_E and A_O are the area of corresponding signals B, A, F2, F1, D2, D1, E and O in each sample, indicated in Table 1 and shown in Figure 1.

1.2. Hydrolysis products in stored samples

The number of moles (N) of fatty acids (FA) and all the glycerides (triglycerides TG%, 1,2-diglycerides 1,2-DG%, 1,3-diglycerides 1,3-DG%, 2-monoglycerides 2-MG%, 1-monoglycerides 1-MG% and glycerol Gol%) present in the lipid samples were expressed as follows:

$$N_{2\text{-}MG} = Pc*A_K/4 \quad \text{[eq.S9]}$$

$N_{1\text{-}MG} = Pc*A_L$ [eq.S10]

$N_{1,2\text{-}DG} = Pc*(A_{I+J}\text{-}2A_L)/2$ [eq.S11]

$N_{TG} = Pc*(2A_{4.26\text{-}4.38}\text{-}A_{I+J}+2A_L)/4$ [eq.S12]

$N_{1,3\text{-}DG} = Pc*(A_{4.04\text{-}4.38}\text{-}2A_{4.26\text{-}4.38}\text{-}2A_L)/5$ [eq.S13]

$N_{FA} = (Pc*(A_A+A_B)\text{-}9N_{TG}\text{-}6N_{1,2\text{-}DG}\text{-}6N_{1,3\text{-}DG}\text{-}3N_{1\text{-}MG}\text{-}3N_{2\text{-}MG})/3$ [eq.S14]

$N_{Gol} = (N_{FA}\text{-}N_{1,2\text{-}DG}\text{-}N_{1,3\text{-}DG}\text{-}2N_{2\text{-}MG}\text{-}2N_{1\text{-}MG})/3$ [eq.S15]

where Pc is the proportionality existing between the area of the ^{1}H NMR signals and the number of protons that generate them, A_K, A_L, A_{I+J}, A_A and A_B are the areas of the corresponding signals indicated in Table 1 and Figure 2, and $A_{4.04\text{-}4.38}$ and $A_{4.26\text{-}4.38}$ represent the area of the signals at 4.04-4.38 ppm and at 4.26-4.38 ppm, respectively.

Using these equations, the molar percentages of the different kinds of glycerides in relation to the total number of moles of glyceryl structures present (NT_{GS}) were determined by using the following equations:

$NT_{GS} = N_{TG}+N_{1,2\text{-}DG}+N_{1,3\text{-}DG}+N_{2\text{-}MG}+N_{1\text{-}MG}+N_{Gol}$ [eq.S16]

$TG\% = 100N_{TG}/NT_{GS}$ [eq.S17]

$1,2\text{-}DG\% = 100N_{1,2\text{-}DG}/NT_{GS}$ [eq.S18]

$1,3\text{-}DG\% = 100N_{1,3\text{-}DG}/NT_{GS}$ [eq.S19]

$2\text{-}MG\% = 100N_{2\text{-}MG}/NT_{GS}$ [eq.S20]

$1\text{-}MG\% = 100N_{1\text{-}MG}/NT_{GS}$ [eq.S21]

$Gol\% = 100N_{Gol}/NT_{GS}$ [eq.S22]

1.3. Omega-3 acyl groups or fatty acids in samples after storage

The molar percentage of total omega-3 acyl groups or fatty acids (ω3%) in relation to the total number of moles of acyl groups (AG) plus fatty acids (FA) present (NT_{AG+FA}) in the lipid samples were estimated as follows:

$NT_{AG+FA} = Pc*(A_A+A_B)/3$ [eq.S23]

$\omega3\% = 100*(Pc*A_B/3)/NT_{AG+FA}$ [eq.S24]

where A_B is the area of the methylic protons of all the omega-3 AG and FA.

1.4. Oxidation products in stored samples

The concentration of saturated aldehydes (alkanals), expressed as millimoles per mol of total AG+FA present, was estimated by using the following equation:

$$\text{Alkanals (mmol/mol AG+FA)} = 1000*(Pc*A_{9.75})/NT_{AG+FA} \qquad \text{[eq.S25]}$$

where $A_{9.75}$ is the area of signal **a** of the aldehydic proton of alkanals at 9.75 ppm, shown in Table 1.

REFERENCES

Guillen, M.D.; Carton, I.; Goicoechea, E.; Uriarte, P.S. Characterization of cod liver oil by spectroscopic techniques. New approaches for the determination of compositional parameters, acyl groups, and cholesterol from ^{1}H nuclear magnetic resonance and Fourier transform infrared spectral data. *J Agric Food Chem* **2008**, *56(19),* 9072-9079. https://doi.org/10.1021/jf801834j

Martinez-Yusta, A.; Goicoechea, E.; Guillen, M.D. A review of thermo-oxidative degradation of food lipids studied by ^{1}H NMR spectroscopy: influence of degradative conditions and food lipid nature. *Compr Rev Food Sci Food Saf* **2014**, *13*, 838-859. https://doi.org/10.1111/1541-4337.12090

Nieva-Echevarría, B.; Goicoechea, E.; Manzanos, M.J.; Guillén, M.D. A method based on ^{1}H NMR spectral data useful to evaluate the hydrolysis level in complex lipid mixtures. *Food Res Int* **2014,** *66*, 379-387. https://doi.org/10.1016/j.foodres.2014.09.031

Nieva-Echevarría, B.; Goicoechea, E.; Guillén, M.D. Polyunsaturated lipids and vitamin A oxidation during cod liver oil *in vitro* gastrointestinal digestion. Antioxidant effect of added BHT. *Food Chem* **2017**, *232*, 733-743. https://doi.org/10.1016/j.foodchem.2017.04.057
